# Novel Local “Off-the-Shelf” Immunotherapy for the Treatment of Myeloma Bone Disease

**DOI:** 10.3390/cells12030448

**Published:** 2023-01-30

**Authors:** Sandra Charvátová, Benjamin Motais, Justyna Czapla, Tomasz Cichoń, Ryszard Smolarczyk, Zuzana Walek, Sebastian Giebel, Roman Hájek, Juli R. Bagó

**Affiliations:** 1 Department of Haematooncology, Faculty of Medicine, University of Ostrava, 70300 Ostrava, Czech Republic; 2 Faculty of Science, University of Ostrava, 70100 Ostrava, Czech Republic; 3 Department of Haematooncology, University Hospital Ostrava, 70800 Ostrava, Czech Republic; 4 Center for Translational Research and Molecular Biology of Cancer, Maria Sklodowska-Curie National Research Institute of Oncology, Gliwice Branch, 44102 Gliwice, Poland; 5 Department of Bone Marrow Transplantation and Onco-Hematology, Maria Sklodowska-Curie National Research Institute of Oncology, Gliwice Branch, 44102 Gliwice, Poland

**Keywords:** immunotherapy, local treatment, cancer, natural killers, myeloma bone disease, fibrin

## Abstract

Myeloma bone disease (MBD) is one of the major complications in multiple myeloma (MM)—the second most frequent hematologic malignancy. It is characterized by the formation of bone lesions due to the local action of proliferating MM cells, and to date, no effective therapy has been developed. In this study, we propose a novel approach for the local treatment of MBD with a combination of natural killer cells (NKs) and mesenchymal stem cells (MSCs) within a fibrin scaffold, altogether known as FINM. The unique biological properties of the NKs and MSCs, joined to the injectable biocompatible fibrin, permitted to obtain an efficient “off-the-shelf” ready-to-use composite for the local treatment of MBD. Our in vitro analyses demonstrate that NKs within FINM exert a robust anti-tumor activity against MM cell lines and primary cells, with the capacity to suppress osteoclast activity (~60%) within in vitro 3D model of MBD. Furthermore, NKs’ post-thawing cytotoxic activity is significantly enhanced (~75%) in the presence of MSCs, which circumvents the decrease of NKs cytotoxicity after thawing, a well-known issue in the cryopreservation of NKs. To reduce the tumor escape, we combined FINM with other therapeutic agents (bortezomib (BZ), and tumor necrosis factor-related apoptosis-inducing ligand (TRAIL)), observing a clear therapeutic synergistic effect in vitro. Finally, the therapeutic efficacy of FINM in combination with BZ and TRAIL was assessed in a mouse model of MM, achieving 16-fold smaller tumors compared to the control group without treatment. These results suggest the potential of FINM to serve as an allogeneic “off-the-shelf” approach to improve the outcomes of patients suffering from MBD.

## 1. Introduction

Multiple myeloma (MM) is a mature B-cell neoplasm defined by the presence of aberrant plasma cells (aPCs) within the bone marrow (BM). Globally, it is the second most frequent hematologic malignancy after leukemia/lymphoma [1]. Typical manifestations of the disease resulting from organ impairment are hypercalcemia, renal insufficiency, anemia, and bone lesions. The outcome of patients with MM has notably augmented as a consequence of new therapeutic approaches such as immunomodulatory drugs, proteasome inhibitors, monoclonal antibodies, and autologous stem cell transplantation [2] with a 10 years survival rate of up to 30–40%. This extension in life expectancy has increased the need for providing long-term supportive care and quality of life for these patients by limiting disease-associated complications.

One of the major disease-associated complications in MM patients is bone disease, which leads to pain, pathological fractures, and mobility issues. Bone pain is present in almost 60% of patients at the time of MM diagnosis, and 90% of them will experience osteolytic lesions [3]. The key factor involved in the pathophysiology of myeloma bone disease (MBD) is the loss of the coupling mechanism between osteoclasts (OCs) and osteoblasts (OBs). In MBD, there is an increase in OCs activity and a reduction in OBs activity, leading to bone resorption [4]. MM cells orchestrate this unbalance in bone remodeling by expressing different cytokines and growth factors that directly or indirectly activate OCs and inactivate OBs. The standard of care for patients with MBD includes treatment with bisphosphonates, radiotherapy, surgical intervention, and filling the defect with bone cement [5,6]. All these different approaches induce the restraining of progressive MBD but are not effective in the healing of bone tissue. Due to the frequency and severity of MBD, new therapeutic approaches are paramount to not only limit the progressive skeletal disability but also to recover the functionality in the bones of these patients.

Recent unprecedented success in cell-based immunotherapy to treat different types of hematological cancer with T cells expressing chimeric antigen receptors (CAR-T), lays the basis for considering cell-based immunotherapy as an efficient alternative for treating MBD [7]. However, CAR-T cell therapy still needs to surpass significant limitations, such as the need for an autologous T cell source to avoid life-threatening complications such as graft-versus-host-disease (GvHD) [8] and the on-target off-tumor toxicity [9]. These limitations have fostered the exploration of other immune cells as a therapeutic vehicle for cancer treatment, such as the natural killer cells (NKs), with exceptional anti-tumor features, low on-target off-tumor toxicity [10], and absence of GvHD in allogenic approaches [11]. Although the therapeutic effect of the NKs to treat MM has been proved in different studies [12,13,14], proper treatment of MBD requires the elimination of the MM cells and the healing of the bone. Another major caveat with the NKs is their high sensitivity to cryopreservation, producing NKs anti-tumor activity loss after thawing [15], limiting, in consequence, their use as a ready-to-use “off-the-shelf” product.

Thus, the optimal treatment of MBD with NKs should include the capacity to regenerate the bone and be therapeutically efficient after thawing. To this end, we considered a combined therapy with mesenchymal stem cells (MSCs) to solve both concerns. The MSCs, have osteoinduction and osteogenesis properties [16,17] and thus have the potential to regenerate bone. On the other hand, the MSCs cells can activate NKs when they are both co-cultivated [18]. In addition, because the MSCs are immunogenic by themselves [19], they allow the possibility of considering an allogeneic approach in combination with NKs.

Lastly, because the MBD is localized myeloma, we explore the possibility of a locoregional delivery to increase the retention and persistence of the therapeutic cells in the tumor site, thus reducing the off-target side effects observed in systemic delivery [20,21,22]. To ensure the retention, persistence, and favorable environment for the therapeutic cells to exert anti-tumor response and tissue regeneration, a three-dimensional support was required. Among different clinically approved scaffolds, fibrin sealants derived from human blood possess essential biological and mechanical properties that lead to their application in many surgical practices and show high potential for bone tissue regeneration [23]. TISSEL (Baxter Healthcare Corp., Deerfield, IL, USA), a clinically approved fibrin sealant, permits the rapid formation of fibrin scaffold after mixing thrombin and fibrinogen proteins. Different clinical studies showed the biostability and biocompatibility of TISSEL fibrin glue [24,25], leading to its use for tissue regeneration of skin [26], liver [27], and bone tissue [28], among others.

Here, we describe the generation and characterization of a new “off-the-shelf” composite for the treatment of MBD comprised of fibrin, NKs, and Wharton jelly (WJ)-derived MSCs (altogether also as FINM). For translational reasons, we created a FINM prototype that permits to be cryopreserved without compromising its therapeutic effect after thawing. Besides, because both cell types do not cause GvHD, an allogeneic approach can be considered, increasing their universal access and availability. We proved in vitro the therapeutic benefits of embedding NKs with WJ-MSCs in a fibrin scaffold for the treatment of MBD and their synergistic therapeutic effect when combined with bortezomib (BZ), a first-line approved proteasome inhibitor agent for the treatment of MM, and tumor-necrosis factor-related apoptosis-inducing ligand (TRAIL), a potent cytotoxic agent that selectively induces anti-tumor effect. To clearly assess the therapeutic efficacy, we investigated the anti-cancer efficacy of FINM in a mouse model of MM. These studies are the first of their kind and permit to define a novel local therapeutic approach to treat MBD and other tumor types with similar pathophysiology.

## 2. Material and Methods (FINM)

### 2.1. Primary Cells

Human peripheral blood mononuclear cells (PBMC) were isolated from buffy coat fraction of healthy donors’ peripheral blood (obtained from Blood Center of the Faculty Hospital of Ostrava with ethics committee approval) by density gradient centrifugation (Ficoll Paque PLUS, density 1.077 g/mL, GE Healthcare, Chicago, IL, USA). Prior the procedure, buffy coats were diluted with Dulbecco’s Phosphate Buffered Saline (DPBS; BioWest, Nuaillé, France) containing 2% of EDTA (Sigma-Aldrich, Munich, Germany) and 2.5% of Albutein^®^ (20%; Grifols, Barcelona, Spain) in ratio 1:3. Obtained PBMC were further used for NKs isolation and OCs differentiation. NKs were isolated from PBMC using autoMACS^®^ Pro Separator (Miltenyi, Bergisch Gladbach, Germany) and NK Cell Isolation Kit (Miltenyi, Bergisch Gladbach, Germany) according to original company protocol. Cells were cultured in NK MACS^®^ Basal Medium (Miltenyi, Bergisch Gladbach, Germany) containing 5% of human AB serum (Sigma-Aldrich, Munich, Germany), 0.5% penicillin/streptomycin (P/S; Lonza, Basel, Switzerland), 500 IU/mL IL-2 (PeproTech, London, UK) and 10 ng/mL IL-15 (PeproTech, London, UK) in 75 cm^2^ flask at density 1 × 10^6^ cells/mL. To obtain differentiated OCs, PBMC were seeded in 6 well plate (6 × 10^5^ cells/well) and cultured in MEM Alpha medium (1×; Gibco, Life technologies, Waltham, MA, USA) supplemented with 10% of FBS (BioWest, Nuaillé, France), 1% of Ultraglutamine 1 (Lonza, Basel, Switzerland), 1% of P/S (Lonza, Basel, Switzerland), 0.2% of puromycin (InvivoGen, Toulouse, France), 25 ng/mL of Macrophage colony stimulating factor (M-CSF; PeproTech, London, UK), 50 ng/mL of Receptor Activator of nuclear factor kappa-B ligand (RANKL; PeproTech, London, UK), 5 ng/mL of Transforming growth factorTGF-β (PeproTech, London, UK) and 1 μM dexamethasone (Sigma-Aldrich, Munich, Germany) for 14 days. OCs differentiation was assessed with the Leukocyte TRAP Kit 387-A (Sigma-Aldrich, Munich, Germany) and according to the manufacturer’s protocol.

Bone marrow mononuclear cells (BMMC) were isolated from BM aspirates by density gradient centrifugation (Ficoll Paque PLUS, density 1.077 g/mL, GE Healthcare, Chicago, IL, USA). Abnormal plasmatic cell fraction (primary MM cells) was selected from BMMC with CD138 MicroBeads (Miltenyi, Bergisch Gladbach, Germany) using the autoMACS^®^ Pro Separator (Miltenyi, Bergisch Gladbach, Germany). The procedure was performed according to original company protocol. WJ-MSCs from Wharton Jelly of umbilical cord of fetal origin (PromoCell, Heidelberg, Germany) were cultured in DMEM High Glucose medium (BioWest, Nuaillé, France) supplemented with 20% of FBS (BioWest, Nuaillé, France), 1% of P/S (Lonza, Basel, Switzerland) and 0.2% of puromycin (InvivoGen, Toulouse, France) in 75 cm^2^ flasks at confluency <80%.

### 2.2. Cell Lines

MM cell lines—RPMI 8226 (ATCC CCL-155), U266 (ATCC TIB-196) and MM1.S (ATCC CRL-2974) were cultured in RPMI 1640 medium (BioWest, Nuaillé, France) supplemented with 10% of FBS (BioWest, Nuaillé, France), 1% of Ultraglutamine 1 (Lonza, Basel, Switzerland), 1% of P/S (Lonza, Basel, Switzerland) and 0.2% of puromycin (InvivoGen, Toulouse, France) in 75 cm^2^ flask at density 1 × 10^6^ cells/mL. All media were changed routinely every 2–3 days. Cell viability and cell proliferation curves were determined by LUNA-FL^TM^ Dual Fluorescence Cell Counter (Logos Biosystems, Anyang, Korea, version 1.4.0).

### 2.3. Bortezomib Treatment

3 × 10^6^ MM cells were seeded in T-25 flasks in complete RPMI 1640 medium (BioWest, Nuaillé, France) and treated with BZ (Velcade, Cambridge, MA, USA) with the following concentrations: RPMI—15 nM; MM1S and U266—10 nM. Cells were cultured in the presence of BZ for 72 h. Viable cells were analyzed by flow cytometry and processed for TRAIL cytotoxicity assay.

### 2.4. Flow Cytometry Analysis

To evaluate NKs phenotype, we performed fluorescence-activated cell sorting (FACS) using BD FACSAria^TM^ III Cell Sorter (BD Biosciences-US, San Jose, CA, USA, version 8.0.1). From previously described phenotype panel [29] we used antibodies CD56-APC-Cy7-Vio770 (Miltenyi, Bergisch Gladbach, Germany), CD16-APC (Miltenyi, Bergisch Gladbach, Germany), NKG2A-PE-Vio770 (Miltenyi, Bergisch Gladbach, Germany), NKG2C-PE (Miltenyi, Bergisch Gladbach, Germany) and NKG2D-PerCP-Cy5.5 (Biolegend, San Diego, CA, USA), KIR2D-FITC (Miltenyi, Bergisch Gladbach, Germany). SYTOX^®^ Blue Dead Cell Stain (Invitrogen, Waltham, MA, USA) was used to evaluate cell viability. Non-NK cell lineage markers were analyzed with the following antibodies: CD3-VioBlue (Miltenyi, Bergisch Gladbach, Germany), TCRg/d-VioBlue (Miltenyi, Bergisch Gladbach, Germany), CD14-VioBlue (Miltenyi, Bergisch Gladbach, Germany), and CD19-VioBlue (Miltenyi, Bergisch Gladbach, Germany).

The phenotype of MM primary cells and cell lines was analyzed by FACSAria III (BD Biosciences-US, San Jose, CA, USA, version 8.0.1) using CD19-PECy7 (Beckman Coulter, Brea, CA, USA), CD56-PE (Agilent Dako, Santa Clara, CA, USA), CD38-FITC (Cytognos, Salamanca, Spain), CD138-APC (Sony, San Jose, CA, USA), and CD45-PB (Agilent Dako, Santa Clara, CA, USA) antibody.

The expression of CD262 (TRAIL-R5) and IFN-γ receptor (IFN-γ-R2) was analyzed in BZ treated/not treated MM cell lines using CD262-APC antibody (BioLegend, San Diego, CA, USA) and IFN-γ-R2-APC antibody (R&D Systems, Minneapolis, USA).

### 2.5. Lentiviral Vectors

The lentiviral construct for the constitutive expression of the bioluminescence (firefly luciferase; FLuc) and the red fluorescence (mCherry) reporters was generated by amplifying the cDNA encoding both reporters (#44965; Addgene, Watertown, MA, USA) and cloned in a lentivirus backbone (#12262; Addgene, Watertown, MA, USA) using standard cloning procedures. The coding sequence of the secreted TRAIL (FLT3L-TRAIL fusion protein) was obtained from [30] and the synthetic gene (Eurofins Genomics, Ebersberg, Germany) cloned into a lentiviral vector (#17448; Addgene, Watertown, MA, USA) in front of a 2A cleavage peptide and the GFP coding sequence by Gibson assembly. Lentiviral vectors were prepared with JetPRIME^®^ (Polyplus, Illkirch-Graffenstaden, France) by transient co-transfection of HEK 293FT cells (gift of Prof. Václav Hořejší, Institute of Molecular Genetics, Prague) with helper plasmids pMD2.G (encoding the VSV-G envelope; #12259; Addgene, Watertown, MA, USA) and psPAX2 (packing plasmid; #12260; Addgene, Watertown, MA, USA) + target plasmid. One day prior transfection, the HEK 293T cells were seeded in 10 cm dish (3 × 10^6^ cells/dish) with 10 mL of DMEM High Glucose medium (BioWest, Nuaillé, France) supplemented with 10% of FBS (BioWest, Nuaillé, France). Five hours after the transfection, medium was replaced, and cells were incubated for next 48 h. Subsequently, virus-containing conditioned medium was collected and concentrated by 20 min-centrifugation (4000× *g*) in Amicon^®^ Ultra-15 Centrifugal Filter Unit (Merck Millipore, Tullagreen, Ireland). Obtained virus concentrate was immediately used for cell infection or was stored in −80 °C for later use. Tumor cell lines, NKs and WJ-MSCs-TRAIL were infected with the virus concentrate at a varying multiplicity of infection in culture media containing 8 µg/mL of Polybrene (Sigma-Aldrich, Munich, Germany).

### 2.6. In Vitro 3D Myeloma Bone Disease Model (OsteoLyse^TM^ Assay)

Osteoclasts (1 × 10^4^ cells/well) and RPMI 8226 cells (1 × 10^4^ cells/well) were seeded in 96-well OsteoLyse^TM^ plate (coated with europium-conjugated human collagen; Lonza, Basel, Switzerland) and covered with cryopreserved FINM (2 × 10^5^ NKs + 2 × 10^4^ WJ-MSCs per well) or fibrin alone (control). Wells were filled with medium containing M-CSF and RANKL used for previous osteoclast differentiation (200 μL/well). After 5 days of cultivation, medium samples were collected from each well and the fluorescence analysis of the released europium-labeled collagen fragments was performed according to the original protocol using Infinite^®^ F Plex (Tecan, Männedorf, Switzerland, version 3.9.0.1).

### 2.7. Fibrin Scaffold Formation

Fibrin scaffold containing NKs and WJ-MSCs was prepared with fibrinogen and thrombin contained within the commercial fibrin sealant TISSEEL (Baxter, Vienna, Austria). Fibrinogen and thrombin were diluted before use to working concentration 20 mg/mL and 10 IU/mL, respectively, with DPBS (BioWest, Nuaillé, France). Per one 96-well plate well, cells (2 × 10^5^ NKs + 2 × 10^4^ WJ-MSCs/WJ-MSCs-TRAI) were resuspended in 15 μL of thrombin and after placing into the well, the suspension was covered with 15 μL of fibrinogen. After 5 min in 37 °C, originated fibrin scaffold containing cells was covered with 150 μL of full RPMI 1640 medium (BioWest, Nuaillé, France).

### 2.8. Monitoring Cell Viability within Fibrin Scaffold

NKs labeled with mCherry-FLuc were seeded together with WJ-MSCs in a 96-well white plate with optical bottom within fibrin scaffold as described above. Cells were cultured for 2 weeks, changing half of the medium every 3 days. At D1, D3, D7 and D14, measurement of the bioluminescence (BLI) induced by addition of D-luciferin potassium salt (Goldbio, St. Louis, MO, USA) to final concentration 0.5 mg/mL was performed. BLI peak values were taken from each well (Infinite^®^ F Plex, Tecan, Männedorf, Switzerland, version 3.9.0.1), and cell viability of the NKs (%) was evaluated based on following equation.

Cell viability = [(mean BLI_D1_ − BLI replicate_DX_)/mean BLI_D1_] * 100 (1)

The experiment was similarly repeated with mCherry-FLuc labeled WJ-MSCs and their viability was determined by the same procedure.

### 2.9. FINM Cytotoxic Assay with Multiple Myeloma Cell Lines

Cytotoxic assays were performed in triplicates in flat white 96-well plates with optical bottom (Thermo Scientific, Waltham, MA, USA). In each well, 2 × 10^4^ target tumor cells labeled with mCherry-FLuc were seeded and covered with cryopreserved FINM containing NKs (2 × 10^5^/well) and WJ-MSCs (2 × 10^4^/well). Wells were then filled with 150 μL of full RPMI 1640 medium (BioWest, Nuaillé, France). Cytotoxicity against cancer cells was evaluated after 7 days of culture by measuring BLI induced by addition of D-luciferin potassium salt (Goldbio, St. Louis, MO, USA) to final concentration 0.5 mg/mL. BLI measurement was performed using Infinite^®^ F Plex (Tecan, Männedorf, Switzerland, version 3.9.0.1). The cytotoxicity evaluation was performed on day 7, when a higher drop in tumor cells was registered. BLI peak values were taken from each well, and cell viability (%) was evaluated based on following equation.

Cell viability = BLI replicate sample/mean BLI control * 100(2)

### 2.10. FINM Cytotoxic Assay with Multiple Myeloma Primary Cells

Isolated primary MM cells were labeled with eBioscience^TM^ Calcein AM Viability Dye (Invitrogen, Waltham, MA, USA) according to the manufacture’s protocol. After labeling, cells were seeded in flat black 96-well plate with optical bottom (Thermo Scientific, Waltham, MA, USA) in density 2 × 10^4^ cells/well and covered with cryopreserved FINM containing WJ-MSCs (2 × 10^4^/well) and NKs (2 × 10^5^/well). After 5 min in 37 °C, cells were covered with 200 μL of full RPMI 1640 medium. After 4 h, plates were spinoculated (5 min, 300 g) and 100 μL of medium from each well was collected for fluorescence (FLUO) measurement. FLUO intensity (excitation 495 nm, emission 515 nm) was measured using Infinite^®^ F Plex (Tecan, Männedorf, Switzerland, version 3.9.0.1). Because the cell viability of primary MM cells rapidly dropped after cultivation, longer times assessment of cytotoxicity was not possible. Cell viability (%) was evaluated based on fluorescence intensity of Calcein released into the medium.

Cell viability = mean FLUO control/FLUO replicate sample * 100(3)

### 2.11. IFN-γ Quantification and Cytotoxic Assay

The FINM (2 × 10^5^ NKs + 2 × 10^4^ WJ-MSCs/well) was seeded together with different MM cell lines (2 × 10^4^/well) in a 96-well plate in full DMEM medium (150 μL/well). 100 μL of conditioned medium was collected from each well after 7 days of co-culture. IFN-γ secreted by NKs was quantified by IFN-γ ELISA Kit (Invitrogen, Waltham, MA, USA) following manufacturer’s protocol. Absorbance (450 nm) was measured using the micro-plate reader Infinite^®^ F Plex (Tecan, Männedorf, Switzerland, version 3.9.0.1). IFN-γ concentrations were calculated based on kit’s standard curve measured alongside the samples.

In a 96-well plate, BZ-treated and untreated MM cells were seeded (4 × 10^4^ cells/well) in 100 μL of complete DMEM medium. Both conditions were treated with recombinant human IFN-γ (Peprotech, London, UK; 100 IU/mL) and left incubated for 3 days. BLI peak values were taken from each well (Infinite^®^ F Plex, Tecan, Männedorf, Switzerland, version 3.9.0.1), and cell viability (%) was calculated using following equation.

Cell viability = BLI replicate sample/mean BLI control * 100(4)

### 2.12. TRAIL Conditioned Medium, Quantification and Cytotoxic assay

The FINM (2 × 10^5^ NKs + 2 × 10^4^ WJ-MSCs-TRAIL/well) was cultivated for 7 days in a 96-well plate in full DMEM medium (150 μL/well). 100 μL of conditioned medium was collected at the end of the cultivation from each well and TRAIL secreted by WJ-MSCs-TRAIL was quantified by TRAIL ELISA Kit (Invitrogen, Waltham, MA, USA) following manufacturer’s protocol. Absorbance (450 nm) was measured using micro-plate reader Infinite^®^ F Plex (Tecan, Männedorf, Switzerland, version 3.9.0.1). TRAIL concentrations were calculated based on kit’s standard curve measured alongside the samples.

In a 96-well plate, BZ-treated and untreated MM cells were seeded at density 4 × 10^4^ cells/well in complete DMEM medium (50 μL/well). Both conditions were treated with conditioned medium containing TRAIL (50 μL/well) and incubated for 3 days. BLI peak values were taken from each well (Infinite^®^ F Plex, Tecan, Männedorf, Switzerland, version 3.9.0.1), and cell viability (%) was calculated using following equation.

Cell viability = BLI replicate sample/mean BLI control * 100(5)

### 2.13. FINM Prototype for In Vivo Treatment

Prototype of an −80 °C-stored “off-the-shelf” product was prepared in the form of two separate components, each having a volume of 100 μL:

Component A—86 μL of human AB serum (Sigma-Aldrich, Munich, Germany), 4 μL of thrombin (250 IU/mL; TISEEL, Baxter, Vienna, Austria), 10 μL of dimethyl sulfoxide for cell culture (DMSO; Panreac AppliChem, Darmstadt, Germany) and cells (1.34 × 10^6^ NKs + 1.34 × 10^5^ WJ-MSCs-TRAIL).

Component B—100 μL Fibrinogen (45.5 mg/mL; TISSEEL, Baxter, Viena, Austria).

Vials with prototype were frozen down (−80 °C) using CoolCell^TM^ cell freezing container (Corning, Corning, NY, USA). For the in vivo treatment of mouse models of MM, each mouse received 500 μL of component A, and 200 μL of component B, locally delivered to the tumor using two separated syringes.

### 2.14. Xenograft Mouse Model

All in vivo experiments were approved by Animal Care Committee and were performed at the Maria Sklodowska-Curie National Research Institute of Oncology (Gliwice, Poland). In total, 25 adult 6–8 weeks old female severe combined immunodeficient mice (Charles River Laboratories, Wilmington, MA, USA) were used. Tumors were formed by right rear flank subcutaneous injection of 7 × 10^6^ RPMI 8226 cells (labeled with mCherry-FLuc) resuspended in a 1:1 mixture of DPBS and Matrigel (Corning, Corning, NY, USA) with total volume of 200 μL. Mice were divided into five groups (5 mice/group) obtaining particular treatment at prescribed day after tumor implantation at D1 (Table 1).

On D7, D8, D14, D21 and D28, bioluminescence imaging was performed in each group to monitor the tumor growth using IVIS Lumina XR In Vivo Imaging System (PerkinElmer, Waltham, MA, USA). Each mice obtained intra-peritoneal injection of D-luciferin (1.5 mg) resuspended in DPBS (100 μL) 10 min before measurement. Exposure time was adjusted based on the obtained signal in each case. Photon emission was quantified by measuring average radiance (p/s/cm^2^/sr) using LivingImage software (PerkinElmer, Waltham, MA, USA; version 4.1.0.11858). After the measurement, mice were returned to the cages, where they were observed for a several minutes. Mice with weight loss over 20% of their initial weight or signs of suffering were sacrificed.

### 2.15. Statistical Analysis

Obtained data were analyzed using GraphPad Prism 5.00 for Windows (San Diego, CA, USA). Student’s two-tailed *t*-test and one-way ANOVA were used to determine statistical significance (*p* < 0.05).

## 3. Results

### 3.1. Concept of Novel Local Immunotherapy for Myeloma Bone Disease—NK Cells and WJ-Mesenchymal Stem Cells as a Therapeutical Cells within the Fibrin Scaffold (FINM)

**Figure 1:**
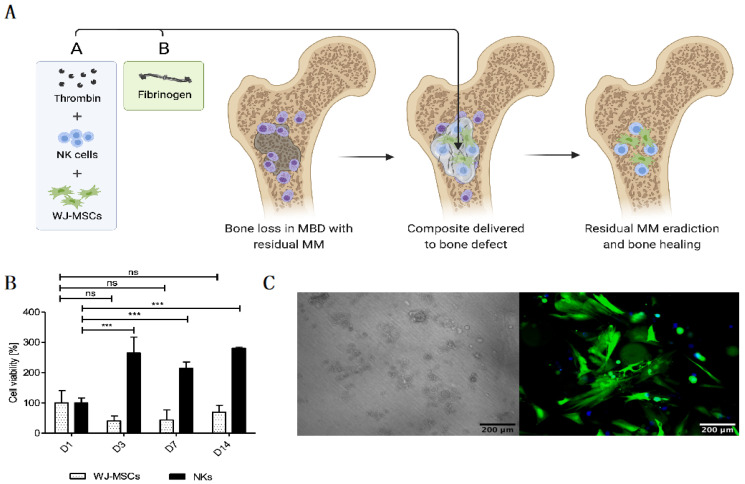
Novel “off-the-shelf” local immunotherapy for myeloma bone disease treatment combining NKs and WJ-MSCs within the fibrin matrix (FINM). (**A**) Schematic drawing of novel local immunotherapy concept leading to myeloma lesions eradication and bone healing after delivery of FINM. (**B**) Viability of WJ-MSCs and NKs at different days (D1, D3, D7, D14) after embed (D0) in fibrin scaffold. (**C**) Fluorescence microscopic image of WJ-MSCs expressing green fluorescence protein (green) within fibrin matrix together with NKs expressing cyan fluorescence protein (blue), at D14 after seeding (scale bar = 200 µm). Data in (**B**) are presented as means ± SD from three technical replicates. ns—not significant; *** −*p* < 0.001; one-way ANOVA, repeated measures test and Student’s *t*-test.

In an attempt to create an optimal “off-the-shelf” local cell-based therapy to treat MBD, we developed the FINM prototype that can be stored frozen and rapidly applied to the treated area after the gentle thawing and mixing of the two components that form the FINM composite: (1) solution A containing activated peripheral blood-derived NKs and WJ-MSCs, cryopreserved in a solution of human albumin, DMSO, and thrombin; and (2) solution B containing fibrinogen. Once both solutions are mixed, a rapid hydrogel of fibrin is formed, permitting the retention of the therapeutic cells in the region of interest (Figure 1A).

The FINM prototype was initially evaluated in an in vitro environment where the viability of both therapeutic cell types (NKs and WJ-MSCs) was assessed within the fibrin matrix. Cell viabilities were tracked over time after embedding in fibrin in a ratio of 10:1 (NKs to WJ-MSCs) at D0. In preliminary experiments, we determined the ratio 10:1 as optimal to achieve a good anti-tumor effect (data not shown). Regarding the NKs, as shown in Figure 1B, the viability was almost three-fold higher on D3, D7 and D14 compared to D1, indicating a significant cell proliferation on the first three days of culture within the FINM. On the other hand, the WJ-MSCs showed a slight decrease in viability on D3 and D7, followed by an increase on D14, achieving on this day similar viability as on D1. Cell viability was calculated following the Equation (1).

The results were in accordance with the observation by fluorescence microscope, where we detected fully viable labeled cells on D14 (Figure 1C). The concentration of fibrinogen and thrombin was adjusted to 20 mg/mL and 10 UI/mL, respectively, to permit retention of the cells as well as to allow the cell migration into the immediate environment [31].

### 3.2. In Vitro Cytotoxic and Anti-Osteolytic Effect of the FINM in Multiple Myeloma

**Figure 2:**
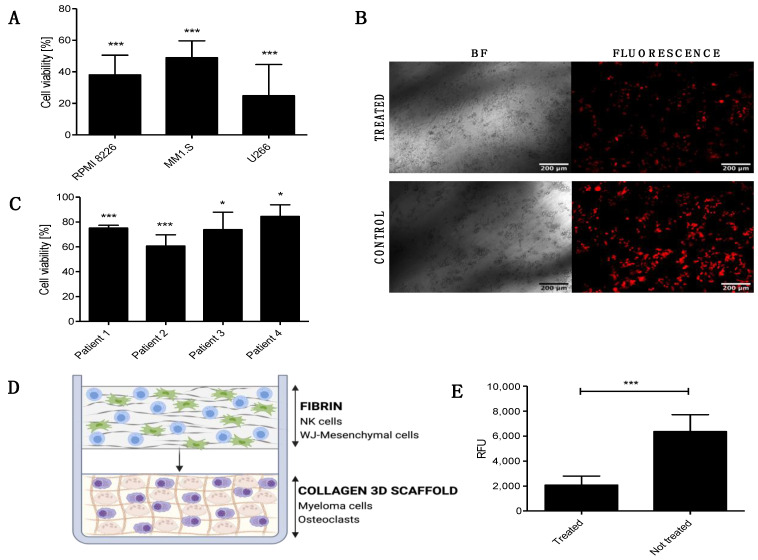
In vitro cytotoxic and anti-osteolytic effect of the FINM prototype in multiple myeloma. (**A**) Viability of RPMI 8226, MM1.S and U266 cells after 7-day co-culture with FINM prototype. (**B**) Fluorescence microscopy image of the RPMI 8226 cells (red), after seven day in co-culture with the FINM prototype (treated) and without (control). (**C**) Viability of primary MM cells from four different patients after 4 h co-culture with the FINM prototype. (**D**) Schematic picture of the prototype application in an in vitro 3D model of MM-induced osteolysis. (**E**) Fluorescence emission (RFU) obtained from supernatant of treated and not treated in vitro model of MM-induced osteolysis, corresponding to cleavage of europium-labelled collagen by OCs. Data in (**A**,**C**,**E**) are presented as means ± SD from three technical replicates. ns—not significant; * −*p* < 0.01; *** −*p* < 0.001; one-way ANOVA, repeated measures test and Student’s *t*-test.

Next, we studied the anti-tumor capability of the FINM against different MM cell lines (RPMI 8226, MM1.S, U266). To this end, we performed an in vitro bioluminescence-based assay [12] where FINM was co-cultured with different MM cell lines stably transduced to express luciferase, and bioluminescence read on day seven. A volume of FINM that led to 10:1 effector-to-target ratio (NKs to MM cells) was used. As shown in Figure 2A, we detected a statistically significant reduction in all MM cell lines tested after co-culture with FINM, with more than 50% less tumor cells, compared to the control without FINM. Cell viability was calculated following the Equation (2). To validate the bioluminescence results, fluorescence images were obtained from treated and not treated wells. Figure 2B shows representative fluorescence images of RPMI 8226 cells (red) after seven days of culture with and without FINM.

To evaluate the translational relevance of the FINM for the local treatment of patients with MBD, we studied in vitro its cytotoxic capacity against primary MM cells from four newly diagnosed MM patients. As shown in Figure 2C, the FINM prototype exerted statistically significant cytotoxicity against all four samples after 4 h of co-cultivation. In this case, the tumor cell viability was calculated using the Calcein AM viability assay, and following the Equation (3).

The induction of osteolysis through the stimulation of the OCs by MM cells is one of the key factors that lead to MBD. Therefore, our next goal was to verify whether the FINM prototype could reduce bone resorption by attenuating OCs activity. To this end, we created an in vitro model that mimicked the pathological bone microenvironment with OCs and MM cells (RPMI 8226) embedded in europium-labeled 3D collagen matrix (OsteoLyse^TM^ Assay Kit, Lonza, Walkersville, MD, USA) (Figure 2D). The labeled collagen permits the calculation of resorption activity and hence the OCs activity by measuring the fluorescence of the supernatant containing fragments of collagen cleaved by OCs. Based on this fluorescence analysis, we evaluated the degree of OCs activity after co-cultivation with the FINM in the 3D MBD model. Interestingly, the results showed more than three times reduction in collagen degradation in treated wells with FINM compared to untreated.

Taken together, these data demonstrate the cytotoxic activity of the FINM against different MM cell lines and primary MM cells, as well as their potential to restore the unbalance bone resorption in MBD by significantly reducing the OCs activity.

### 3.3. Synergistic Effect in Combinatorial Therapy of Bortezomib with FINM

**Figure 3:**
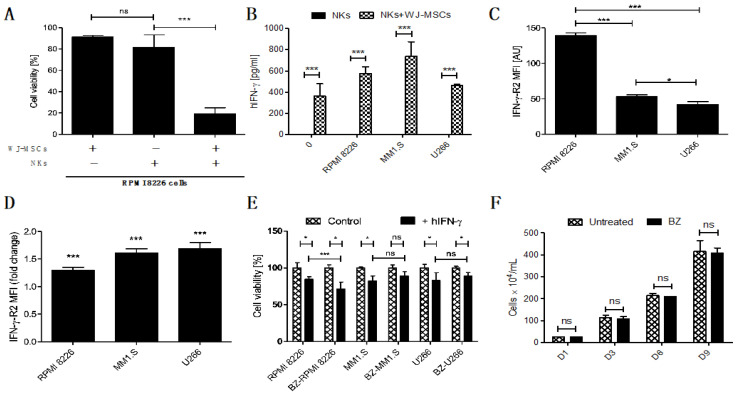
Prominence of the IFN-y in FINM and its potential in treatment of MM in combination with BZ. (**A**) Viability of RPMI 8226 cells after 7-day co-culture with FINM prototype containing WJ-MSCs/NKs/combination. (**B**) IFN-y produced by NKs in seven day co-culture with MM cell lines +/− WJ-MSCs in fibrin covered with culture medium. (**C**) Mean IFN-y-R2 expression per cell (MFI) in RPMI 8226, MM1.S and U266. (**D**) Fold change in mean IFN-y-R2 expression per cell (MFI) in RPMI 8226, MM1.S and U266 cells in culture with BZ. (**E**) hIFN-y effect on cell viability of the BZ-pretreted (BZ-) and not pretreated MM cells (RPMI 8226, MM1.S and U266). (**F**) RPMI 8226 cell line growth in culture with/without BZ (nine days). Data in (**A**–**F**) are presented as means ± SD from three technical replicates. ns—not significant; * −*p* < 0.01; *** −*p* < 0.001; one-way ANOVA, repeated measures test and Student’s *t*-test.

To properly evaluate the synergistic anti-tumor effect of embedding NKs with WJ-MSCs, we studied the cytotoxicity of the FINM against MM cell line RPMI 8226 without WJ-MSCs or NKs. The results obtained show a clear synergistic effect when the FINM combines NKs with WJ-MSCs, with nearly four-fold lower tumor cell viability compared to FINM with NKs alone after seven days in co-culture (Figure 3A). This synergistic effect could be explained, at least partially, by the increase in IFN-y production by NKs that are in contact with WJ-MSCs, as is suggested by Heike Thomas et al. [18]. In this study, they indicate that MSCs-prime NKs to have a higher sensitivity for IL-12 by a still unknown mechanism, producing the stimulation of NKs mediated IFN-y production and secretion.

To confirm this probable mechanism in the FINM, we quantified the amount of IFN-y in the co-culture of the FINM with and without WJ-MSCs, as well as in co-culture with different MM cell lines (RPMI 8226, MM1.S, U266). After seven days of co-cultivation, and in concordance with the study of Heike Thomas et al., we observed a significantly higher amount of IFN-y in FINM containing WJ-MSCs + NKs, compared to FINM with only NKs or only WJ-MSCs (data not shown and equal to 0). This significant increase in IFN-y production was observed in all FINM with WJ-MSCs + NKs, independently of the presence of the tumor cells (Figure 3B). To understand the possible effect of this increase in IFN-y secretion against MM cells, we confirmed the expression of the IFN-y receptor (IFN-y-R2) in RPMI 8226, MM1.S, and U266 cell lines by flow cytometry analysis (Figure 3C). Besides, we observed a statistically significant reduction of the MM cell viability in all three tumor cell lines after cultivation with human IFN-y (hIFN-y) for 72 h, compared to controls without hIFN-y (Figure 3E).

Because the standard of care of patients with MM includes the administration of proteasome inhibitors, such as BZ, we studied a potential synergistic effect in combination with FINM. For this purpose, we studied the change in expression of IFN-y-R2 in MM cell lines after treatment with BZ. Interestingly, the treatment of MM cell lines with BZ significantly increased the mean expression of IFN-y-R2 per cell, as shown in Figure 3D. Because of the high expression of IFN-y by FINM prototype, these results corroborated the potential synergistic effect of combining BZ with FINM due to increase of IFN-y-R2 in MM cells after BZ treatment. This potential synergistic effect was verified by comparing the cell viability of MM cell lines after treatment with BZ and cultivated with IFN-y (Figure 3E). Cell viability was calculated following the Equation (4). The results obtained showed a statistically significant reduction in RPMI 8226 cells viability in combinatorial therapy of BZ with hIFN-y, compared to controls with single treatment of hIFN-y. In contrast, no significant reduction in cell viability was observed in MM1.S and U266 cell viability after co-cultivation with BZ and hIFN-y, compared to controls with single treatment of hIFN-y. These synergistic effect differences between MM cell lines could be caused by the disparity in the expression of IFN-y-R2 per cell after treatment with BZ, with clear synergistic effect in RPMI 8226 which express higher IFN-y-R2 per cell after treatment BZ, compared to no synergistic effect in MM1.S and U266 which express lower IFN-y-R2 per cell after treatment with BZ. To discard the possibility that the synergistic effect observed in RPMI 8226 is merely caused by BZ effect in cell proliferation, we compared the cell growth over time of RPMI 8226 treated and not treated with BZ. As shown in Figure 3F, we did not observe any difference between the two groups over time, dismissing the possibility that the synergistic effect is a consequence of the BZ effect on RPMI 8226 proliferation.

Together, these results show the therapeutic synergistic effect of combining NKs and WJ-MSCs in FINM prototype, as well as the potential of combining FINM with BZ to increase the cytotoxic effect against MM cells.

**Figure 4:**
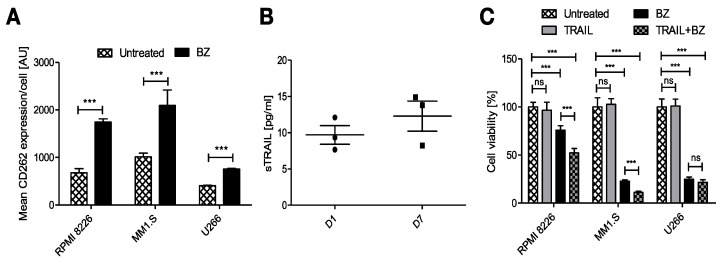
Potential combinatorial therapy against MM with engineered FINM for the expression of TRAIL with BZ. (**A**) Mean CD262 expression per cell (MFI) in RPMI 8226, MM1.S and U266 cells in culture with/without BZ. (**B**) Expression of soluble TRAIL protein by engineered WJ-MSCs within FINM. (**C**) MM cell lines viability with combinatorial and single-agent treatment. Data in (**A**–**C**) are presented as means ± SD from three technical replicates. ns—not significant; *** −*p* < 0.001; one-way ANOVA, repeated measures test and Student’s *t*-test.

Intending to reduce the tumor escape after the treatment with FINM, we studied other potential combinatorial therapies. After cultivating the MM cell lines with BZ, we also observed a notable increase in the average expression of the CD262 per cell (Figure 4A). This receptor, also known as TRAIL receptor 2 or death receptor, is a receptor of the tumor necrosis factor receptor superfamily that binds to TRAIL and mediates apoptosis. Because CD262 is mainly over-expressed in tumor cells, it becomes an ideal target against tumor cells. For this reason, and because of the potential synergistic effect with BZ, we genetically modified WJ-MSCs for the constitutive expression of TRAIL. These engineered WJ-MSCs were able to produce 9.7 ± 2.25 pg/mL of TRAIL after 24 h embedded within FINM (96-well plate with 2 × 10^4^ WJ-MSCs-TRAIL, 2 × 10^5^ NKs, 30 μL of fibrin), with a slight increase after seven days of culture (Figure 4B).

We next studied the anti-tumor effect of combining BZ with secreted TRAIL from the FINM. The three MM cell lines viability was tracked after their treatment with BZ and cultivation with conditioned medium containing TRAIL from engineered FINM and compared to the cell viability obtained with single treatments. Cell viability was calculated following the Equation (5). As shown in Figure 4C, the cell viability between the different groups after three days of culture revealed a synergistic anti-tumor effect in combinatorial therapy with BZ and TRAIL, with statistically significant lower cell viability compared to single treatments against RPMI 8226 and MM1.S. On the other hand, this synergistic effect was not observed in combinatorial therapy against U266, most likely due to the lower increase in expression of CD266 after treatment with BZ.

Collectively, the data obtained verify the therapeutic synergistic effect of combining engineered FINM for the expression of TRAIL with BZ.

### 3.4. Efficacy of FINM in Combination with Bortezomib in MM Mouse Model

**Figure 5:**
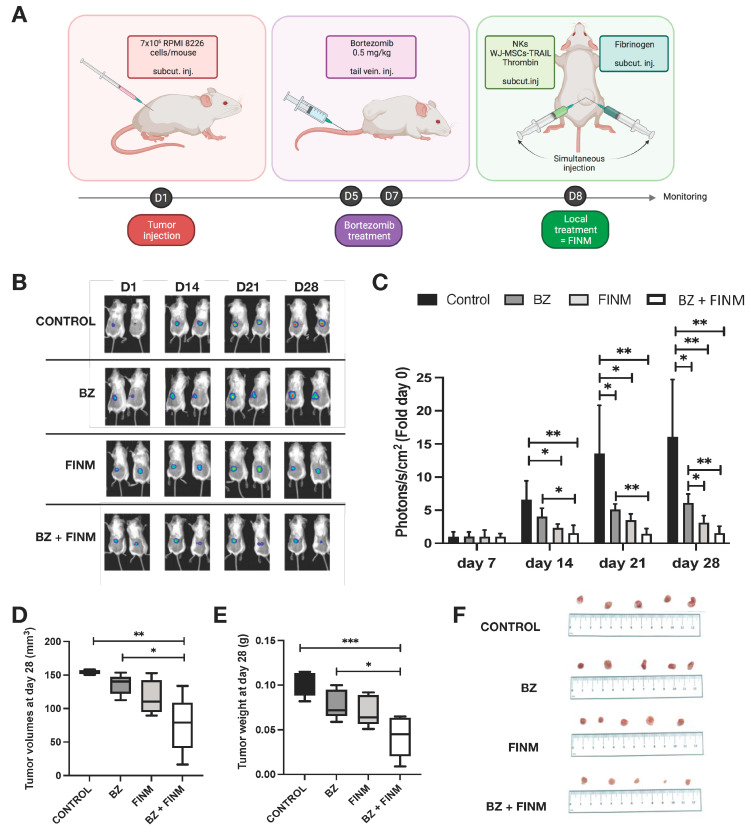
FINM efficiency in in vivo mouse model. (**A**) Schematic description of the experiment including RPMI 8226 cells inoculation, Bortezomib treatment (BZ) and local treatment with FINM prototype expressing TRAIL (FINM). (**B**) Representative bioluminescence images in each group at D7, D14, D21 and D28 after RPMI 8226 cell inoculation (D1). (**C**) Tumor bioluminescence (photon flux) at D7, D14, D21 and D28 after inoculation. (**D**) Tumor volumes in different treatment groups at D28. (**E**) Tumor weights in different treatment groups at D28. (**F**) Image of the tumors retrieved in each group at D28. Data in (**C**–**E**) are presented as means ± SD from three technical replicates. Ns—not significant; * −*p* < 0.01; ** −*p* < 0.005; *** −*p* < 0.001; one-way ANOVA, repeated measures test and Student’s *t*-test.

For the in vivo evaluation of the therapeutic efficacy of the FINM treatment, we used mice bearing a subcutaneous MM model with RPMI 8226 cells genetically modified for constitutive expression of luciferase. Based on our previous in vitro evidence, we tested in those mice the therapeutic effect of combining FINM (genetically engineered for the expression of TRAIL) with BZ. The treatment time points, the type of treatment, and the route of administration is depicted in Figure 5A. By bioluminescence tracking of the tumor progression in each treatment group (Control; BZ; FINM; FINM + BZ), we compared the effect of each agent and evaluated the synergy of systemically administered BZ with local cell-based immunotherapy treatment (FINM). Serial bioluminescence imaging showed that single and combined treatment attenuated the tumor progression compared to control without treatment (Figure 4B). Combined therapy with FINM and BZ had a significantly higher therapeutic effect compared to single treatment with only FINM or BZ, achieving on D28 six-fold smaller tumors compared to single treatment with BZ, and 16-fold smaller tumors compared to control group (not treated). These imaging results were confirmed by post-mortem retrieval of the individual tumors from each group on D28 (Figure 5D), following volume (Figure 5E), and weight calculations (Figure 5F).

These results support the conclusion that FINM has a strong effect against MM cells, and their anti-tumor properties can be boosted by combining with BZ and TRAIL.

## 4. Discussion

Cell-based immunotherapy has revolutionized the cancer treatment, changing the landscape of oncology by substantially prolonging the survival of patients [32]. However, its conventional systemic delivery of therapeutic cells has several drawbacks. One of them is the insufficient tumor-homing of the therapeutic cells [33], which results in insufficient therapeutic effect even with repeated cycles of therapy. Another major drawback is the off-target side effect that occurs when the therapeutic cells start attacking healthy tissue away of the tumor foci [34]. Recent studies showed that these issues could be overcome by delivering the therapeutic cells directly to the tumor site. Thus, increasing the number of therapeutic cells within the tumor site by reducing the migration distance, and diminishing off-target side effects [35]. In this line, we proposed a novel effective local immunotherapy to treat MBD with the potential to become an “off-the-shelf” product. With this aim, we searched for an allogenic approach that permits universal use, rapid availability and effective in eliminating tumor cells and regenerating the bone. To this end, we created a prototype (FINM) consisting of three components: (1) NKs—innate immune cells with strong cytotoxic activity against tumor cells; (2) WJ-MSCs—multi-potent stem cells capable of connective tissue regeneration and stimulation of NKs activity; and (3) fibrin—biocompatible scaffold for the retention of the therapeutic cells, providing a favorable environment for bone regeneration.

From the different NKs sources, we decided to use peripheral blood derived NKs for two main reasons. First, compared to other cell sources, such as umbilical cord blood [36] and hematopoietic stem cells [37], a large number of NKs can be collected from a single donor, allowing us to attain clinically relevant cell numbers using a simple expansion procedure we developed previously [12]. Second, unlike NKs derived from induced pluripotent stem cells [38,39], there is no risk of teratoma formation.

The MSCs are cells with unique features, such as multi-potency, the ability of multi-lineage differentiating, and immunomodulatory capacity. Because of these unique properties, they have been utilized in various medical fields, such as tissue regeneration [40] or cancer treatment [41]. Regarding the MSC sources, we decided to explore the use of WJ-MSCs harvested from umbilical cord tissue because they are more abundant than MSCs derived from bone marrow and adipose tissue, they can be obtained in a non-invasive manner, and they are considered to have the most potential for tissue regeneration [42].

Fibrin, an insoluble fibrous protein with various in vivo functions [43], is already commonly used in clinical practice, most often as a sealant [44]. Its most significant advantages are its easy handling, including the possibility of injection in liquid form and biocompatibility [45]. Our testing showed that fibrin work as an appropriate extracellular matrix. At a suitably selected concentration [31], fibrin is able to retain therapeutic cells in the site of interest while allowing them to migrate toward the tumor cells, where they can exert their effector function.

To permit a ready-to-use product, we cryopreserved the three FINM components in two solutions. One solution with the therapeutical cells (NKs, WJ-MSCs), human albumin, DMSO, and thrombin, and another solution containing fibrinogen. Once both solutions were thawed and combined, the mixture was able to immediately form a fibrin scaffold containing highly viable NKs and WJ-MSCs, with a long-term viability (>2 weeks) within the fibrin environment and, in the case of NKs, also significant proliferation. The NKs also showed potent cytotoxicity against the MM cell lines—RPMI 8226, MM1.S, and U266, as well as against the primary MM cells obtained from the BM aspirates of the patients with MM. This cytotoxicity was not observed in the case of FINM without WJ-MSCs, suggesting that the WJ-MSCs serve as an NKs activator after thawing. This finding circumvents one of the main problems that could face the cryopreserved FINM and corresponding to the loss of cytotoxicity after thawing. A recent study points out that this can be the cause of a persistent failure of clinical trials in which NKs therapy is used for treating solid tumors [15]. There are contradictory studies regarding the effect of MSCs on NKs, with some authors reporting a suppressive effect of the MSCs on NKs [46,47] cytotoxicity, and other authors observing immuno-stimulatory properties from the MSC towards the NKs [18,48]. These last authors, in line with our results, showed that MSCs-primed NKs to increase the expression of IFN-y. Our data obtained from co-culture experiments clearly show strong immuno-stimulatory effect of WJ-MSCs on IL-2 + IL-15 activated NKs, which lead to increased cytotoxicity against MM cells. Based on our ELISA results, we link the observed effect to the enhanced production of the IFN-y, which itself exert an anti-proliferation effect on MM cells expressing IFN-y receptor.

To reduce the tumor escape after the treatment with FINM, we studied the possibility of combining it with other therapeutic agents, such as the proteasome inhibitor BZ. It was approved for the treatment of MM in 2003 and became an established component of combinatorial therapies against MM [49]. In addition to its direct pro-apoptotic effect [50], BZ shows the ability to make MM cells more sensitive to immune cells [51]. Our in vitro data show that tested MM cell lines up-regulate the expression of CD262 and IFN-y-R2 as a response to BZ action. Due to the high production of IFN-y by NKs embedded in FINM, the systemic use of BZ together with local application of FINM appear to be therapeutically advantageous. This potential synergistic effect was verified in vitro, where we observed a statistically significant reduction in RPMI 8226 cells viability in combinatorial therapy of BZ with hIFN-y, compared to controls with single treatment of hIFN-y. This synergistic effect was not observed in MM1.S and U266, most probably due to their low expression of IFN-y-R2 per cell after treatment with BZ, compared to high expression of IFN-y-R2 observed in BZ-treated RPMI 8226 cells. Because we also observed a notable increase in the average expression of the CD262 per cell in MM cell lines after treatment with BZ, we explored the possibility of combining the treatment of BZ + FINM with the expression of TRAIL, which can induce the apoptosis in TRAIL-R (CD262) expressing cells, mostly tumor cells [52] including MM cells [53]. We purposefully chose to express TRAIL in WJ-MSCs because they can be easily genetically modified [54], and a variety of studies have shown their potential to release TRAIL and serve as a robust therapeutic against different types of cancer [24,55]. In vitro, the combination of BZ + FINM with WJ-MSCs-mediated secretion of TRAIL markedly reduces the viability of the different MM cell lines, with statistically significant lower cell viability compared to single treatments against RPMI 8226 and MM1.S, but not against U266, most likely due to its low expression of CD266 after treatment with BZ.

In vivo, we evaluated the therapeutic efficacy of FINM expressing TRAIL in a mouse model of MM. To mimic the clinical MM patient therapy, we delivered the FINM once the MM was consolidated (eight days after implantation). We found a statistically significant inhibition in MM growth in mice with a combined therapy (FINM and BZ), compared to single treatment with only BZ (six-fold smaller tumors) and control without treatment (16-fold smaller tumors). Despite observing a potent anti-MM effect of FINM in combination with BZ, complete tumor suppression was not achieved, most probably because we only performed one FINM injection. In an effort to address this concern, we will explore in the future the possibility of completely eradicate the tumor increasing the number of injections.

## 5. Conclusions

In vitro and in vivo findings presented in this study demonstrate the effectivity of novel local cancer immunotherapy, which connects the properties of fibrin, NKs and WJ-MSCs expressing the cytotoxic agent TRAIL (together FINM). In addition to its therapeutic efficacy, the FINM provide two major medical needs; an allogeneic cell source and a cryopreserved product that permits a ready-to-use, “off-the-shelf” therapeutic approach to treat MBD. Future studies will focus on maximizing the killing of residual MM cells by increasing the FINM injections or testing new combinatorial therapies such as bisphosphonates, radiotherapy and bone cement, as well as exploring their therapeutic efficacy in other types of tumors with similar physiopathology as the MBD.

## Figures and Tables

**Table 1:** Description of the treatment delivered to mice bearing MM tumors (4 treatment groups-G1–4).

Treatment	Day	INJ. Type	Treatment Composition	G1	G2	G3	G4
Bortezomib	5, 7	Tail vein	100 μL of DPBS containing BZ (0.5 mg/kg)	✓	✓	✕	✕
FINM	8	Subcutaneous peritumoral	Syringe A–500 μL of prototype component A	✓	✕	✓	✕
			Syringe B–200 μlL of prototype component B				

## Data Availability

The data presented in this study are available from the corresponding author upon request.

## Abbreviations

aPCs	Abnormal plasmatic cells
BLI	Bioluminescence
BM	Bone marrow
BMMC	Bone marrow mononuclear cells
BZ	Bortezomib
CD262	Cluster of differentiation 262; Tumor necrosis factor-related apoptosis-inducing ligand receptor 5
D	Day
DMSO	Dimethyl sulfoxide
DPBS	Dulbecco’s phosphate-buffered saline
ELISA	The enzyme linked immunosorbent assay
FACS	Fluorescence-activated cell sorting Multiple myeloma
FLUO	Fluorescence
FINM	Fibrin + Natural killer cells + Mesenchymal stem cells
hIFN-γ	Human Interferon gamma
IFN-γ	Interferon gamma
IFN-γ-R2	Interferon gamma receptor 2
IL	Interleukin
MBD	Myeloma bone disease
MM	Multiple myeloma
MSCs	Mesenchymal stem cells
NKs	Natural killer cells
OBs	Osteoblasts
OCs	Osteoclasts
P/S	Penicillin + streptomycin
PB	Peripheral blood
PBMC	Peripheral blood mononuclear cells
TRAIL	Tumor necrosis factor-related apoptosis-inducing ligand
TRAIL-R	Tumor necrosis factor-related apoptosis-inducing ligand receptor
TRAP	Tartrate-resistant acid phosphatase
WJ-MSCs	Wharton jelly mesenchymal stem cells
